# Microbial co-occurrence network topological properties link with reactor parameters and reveal importance of low-abundance genera

**DOI:** 10.1038/s41522-021-00263-y

**Published:** 2022-01-17

**Authors:** Bing Guo, Lei Zhang, Huijuan Sun, Mengjiao Gao, Najiaowa Yu, Qianyi Zhang, Anqi Mou, Yang Liu

**Affiliations:** 1 Department of Civil and Environmental Engineering, University of Alberta, Edmonton, AB T6G 1H9 Canada; 2 Centre for Environmental Health and Engineering (CEHE), Department of Civil and Environmental Engineering, University of Surrey, Guildford, GU2 7XH UK

**Keywords:** Next-generation sequencing, Microbial ecology

## Abstract

Operational factors and microbial interactions affect the ecology in anaerobic digestion systems. From 12 lab-scale reactors operated under distinct engineering conditions, bacterial communities were found driven by temperature, while archaeal communities by both temperature and substrate properties. Combining the bacterial and archaeal community clustering patterns led to five sample groups (ambient, mesophilic low-solid-substrate, mesophilic, mesophilic co-digestion and thermophilic) for co-occurrence network analysis. Network topological properties were associated with substrate characteristics and hydrolysis-methanogenesis balance. The hydrolysis efficiency correlated (*p* < 0.05) with clustering coefficient positively and with normalized betweenness negatively. The influent particulate COD ratio and the relative differential hydrolysis-methanogenesis efficiency (*D*_efficiency_) correlated negatively with the average path length (*p* < 0.05). Individual genera’s topological properties showed more connector genera in thermophilic network, representing stronger inter-module communication. Individual genera’s normalized degree and betweenness revealed that lower-abundance genera (as low as 0.1%) could perform central hub roles and communication roles, maintaining the stability and functionality of the microbial community.

## Introduction

Anaerobic digestion (AD) is a promising technology for organic waste/wastewater treatment to recover biogas as energy and reduce the risk to environmental and human health^1–3^. The successful AD system depends on robust microbial community, effective microbial symbiosis and balanced hydrolysis, acidogenesis, acetogenesis and methanogenesis, which involve different functional groups. To uncover the AD microbiome “blackbox” and to manage the AD systems, the first task has been deciphering the links between operational factors and microbial community assembly and functionality^4,5^. Previous studies have used the strategy of identifying the most abundant or core microorganisms and correlating them with operational factors and performance to illustrate the key factors and functional microorganisms^1,2,4,6,7^. This approach reflects the selection force on the dominant microorganisms (e.g., top 10, top 20, or >1%) and narrows down the target microorganisms for in-depth genomic and functional analysis. Whereas, the importance and functions of lower-abundance (e.g., 0.1%) members are not fully revealed, and the microbial interactions and ecological roles in the communities remain unclear. Due to the complexity nature of microbiome structure and function, unraveling the microbial interactions within a functional group and between different groups and revealing the microorganisms’ ecological roles and importance in predicting system performances are challenging^8^.

Based on network theory, the co-occurrence of microorganisms can be modeled using network analysis to illustrate microbial relationships and responses to variations of operational factors and suggest clustering of sub-communities. Meanwhile, the network’s topological properties, e.g., modularity and connectivity, may associate with operational and performance parameters and imply the general ecological interaction of the system^9–14^. The topological characteristics of individual microorganisms can be used to infer their ecological roles in the network. Previous studies used microbial communities from different AD systems to construct one global network and link the operational factors to demonstrate their impacts on the microbial eco-system^10,13^. Different feedstock substrates (e.g., waste activated sludge, food waste, manure) have been studied as one important factor affecting the microbial co-occurrence networks for full-scale AD systems^15^. However, operational factors for full-scale systems may vary within and between systems, and pooling all microbial communities fed with different substrates to perform one network analysis may not reveal the correlations between microorganisms properly. Rather, it is rational to build separate networks for each substrate type or each reactor, instead of one network^9,12,13,16^. Very few studies have investigated separated networks to compare different systems. In one study, four separate networks were built for AD reactors treating dairy manure or co-digestion of poultry waste at different organic loading rates and demonstrated that the network topological features and substrate availability were associated^9^. Another study compared four AD networks at different temperatures and found that the topological features were correlated with methane production rate but not temperature^12^.

Multi-network topological characteristic comparison was conducted in other wastewater treatment systems. For example, in activated sludge, the network topological features were mainly associated with substrate characteristics, i.e., the ratio of biological oxygen demand and chemical oxygen demand^17^. In activated carbon biofilter, the seasonal variation and ozonation treatment affected the network properties^18^. While the number of studies on multi-network comparison is limited, there is a need to explore the AD microbial network with appropriate operational parameter constrains to better understand the links between operation, microbial community and system performance^9,18^.

This study aims at investigating the impacts of operational parameters on AD performances and microbial community ecology using blackwater as a feedstock under various conditions. Temperature, reactor design, organic loading rate and substrate characteristics were control variables. In total, 52 microbial samples from 12 reactors were used to analyze microbial community diversity and composition and to construct co-occurrence networks. Separate networks were built for different groups based on the community similarity and the network topological properties were associated with reactor operational and performance variables.

## Results

### Reactor performances

The reactor performances varied at different operational conditions (Table 1). The mesophilic co-digestion, thermophilic co-digestion, and thermophilic reactors showed higher hydrolysis efficiencies (80.5%, 69.1%, 51.8% respectively) than mesophilic (low-solid-substrate, regular-substrate and concentrated-substrate) and ambient conditions (16.0–35.7%). The methanogenesis efficiency also showed higher values in the mesophilic co-digestion (77.1%), thermophilic co-digestion (68.4%), and thermophilic blackwater (51.6%) than the rest (30.8–43.6%). It should be noted that the mesophilic low-solid-substrate condition showed higher methanogenesis efficiency than its hydrolysis efficiencies because that the substrate contained high soluble COD ratio (62% of the total COD), lowering the requirement for substrate hydrolysis.

**Table 1:** Reactor performances and sludge specific methanogenic activities (SMA). Data shown are average values and standard deviation in brackets.

Reactor description	Hydrolysis efficiency	Methanogenesis efficiency	SMA_H_2_	SMA_Acetate	CH_4_ production rate	Eff. tCOD	Eff. sCOD	Eff. pH	Eff. VFA^a^	Eff. TAN^b^	FA^c^
	%	%	gCOD/gVSS/d	gCOD/gVSS/d	gCOD/gVSS/d	g/L	g/L		mg/L	mgN/L	mgN/L
Ambient (A)	35.7 (6.4)	31.7 (10.2)	0.023 (0.00)	0.021 (0.00)	0.025–0.133	NA	0.2 (0.0)	7.0 (0.1)	150 (85)	NA	NA
Mesophilic Low-solid-substrate (M.LS)	16.0 (12.7)	42.8 (17.3)	0.26 (0.06)	0.10 (0.06)	0.005–0.121	0.6 (0.2)	0.5 (0.1)	8.2 (0.2)	41 (38)	961 (378)	194 (89)
Mesophilic regular-substrate (M.reg)	21.4 (8.6)	30.8 (6.2)	0.39 (0.11)	0.18 (0.03)	0.008–0.037	0.2 (0.0)	0.1 (0.0)	7.5 (0.1)	23 (12)	93 (26)	4 (2)
Mesophilic concentrated-substrate (M.conc)	31.4 (1.9)	43.6 (2.6)	0.42 (0.06)	0.14 (0.05)	0.014–0.088	1.3 (0.2)	0.6 (0.0)	7.9 (0.1)	56 (23)	1220 (17)	119 (18)
Mesophilic Co-digestion ^d^ (M.Co)	80.5 (6.8)	77.1 (5.6)	0.18 (0.03)	0.11 (0.02)	0.063–0.213	8.4 (1.5)	4.4 (1.1)	7.3 (0.0)	834 (201)	1319 (0)	36 (0)
Thermophilic (T)	51.8 (4.7)	51.6 (6.8)	0.11 (0.01)	0.06 (0.02)	0.022–0.059	4.9 (0.4)	2.2 (0.3)	7.0 (0.2)	350 (252)	1217 (130)	46 (15)
Thermophilic Co-digestion ^d^ (T.Co)	69.1 (5.7)	68.4 (3.9)	0.34 (0.14)	0.14 (0.02)	0.021–0.090	11.4 (3.3)	7.1 (2.3)	7.7 (0.0)	2493 (2232)	1410 (0)	259 (0)

^a^VFA, volatile fatty acids, acetate, propionate and butyrate.

^b^TAN, total ammonia nitrogen.

^c^FA, free ammonia.

^d^Co-digestion is the blackwater and food waste at a volatile solid mixing ratio of 1:2.

The specific methanogenic activity (SMA) was measured for hydrogenotrophic (SMA_H_2_) and acetoclastic methanogenesis (SMA_Acetate). All reactors showed higher SMA_H_2_ than SMA_Acetate, suggesting that blackwater AD is dominated by hydrogenotrophic methanogenesis rather than acetoclastic methanogenesis, which agrees with the archaeal community composition that hydrogenotrophic methanogens dominated the blackwater AD (Fig. 2B). It can be due to the high content of nitrogen in blackwater and the resulted high total and free ammonia concentrations (93–1410 mg-N/L, free ammonia 4–259 mg-N/L), which leads to inhibition to acetoclastic methanogenesis^1^. The ambient temperature condition resulted in the lowest SMA and the smallest difference between SMA_H_2_ and SMA_Acetate among all reactors due to the lowest temperature and microbial activities.

The effluent total COD concentrations were highest in the thermophilic and mesophilic co-digestion reactors (11.4 g/L and 8.4 g/L, respectively) since their influent COD concentrations were the highest. The thermophilic reactors showed the third highest effluent COD at 4.9 g/L, also due to the high influent COD. The soluble COD and total volatile fatty acids (VFAs) had similar trends with effluent COD. Effluent TAN and free ammonia concentrations were below the inhibitory level^19^.

### Microbial community diversity and composition

**Fig. 1:**
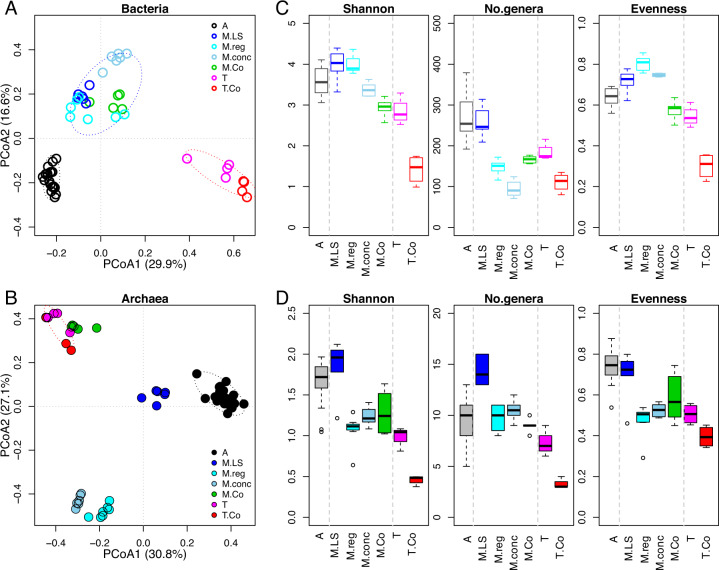
Principal coordinate analysis (PCoA) of Bray–Curtis distance and alpha diversity. **A** PCoA of bacterial communities. **B** PCoA of archaeal communities. **C** Alpha-diversity index of bacterial communities. **D** Alpha-diversity index of archaeal communities. Reactor abbreviation A: Ambient; M.LS: Mesophilic Low-solid-substrate; M.reg: Mesophilic regular-substrate; M.conc: Mesophilic concentrated-substrate; M.Co: Mesophilic Co-digestion; T: Thermophilic; T.Co: Thermophilic Co-digestion. Same sample symbols indicate replicates from the same group.

To compare the microbial community beta-diversity, the Bray-Curtis distance was calculated and ordinated with the Principal Coordinate Analysis (PCoA) for bacterial and archaeal communities separately (Fig. 1) because that archaeal community has been reported to be influenced differently with bacterial community^15,20^. The bacterial communities were clustered into three groups that associated with reactor temperature (ambient, mesophilic and thermophilic, Fig. 1A). This trend reveals that temperature is the key factor that determines the bacterial community composition. ANOSIM analysis showed that both temperature and substrate conditions had a significant effect (*p* < 0.001) on bacterial communities. The PERMANOVA results showed that temperature can explain 43% and substrate can explain 22% of the variance for bacterial community (Table S1).

The archaeal communities showed different clustering patterns with the bacterial communities (Fig. 1B). The ambient and thermophilic reactors still formed their own clusters with a far distance, whereas the mesophilic communities divided into three sub-clusters. The first sub-cluster, consisting of mesophilic co-digestion archaeal communities, was adjacent to the thermophilic archaeal communities. The second sub-cluster, mesophilic low-solid-substrate communities, was closer to the ambient reactor communities. The third sub-cluster includes the regular-substrate and concentrated-substrate reactor samples. Although temperature was an important factor affecting the archaeal community, there were other factors impacting the archaeal community, especially under the mesophilic condition. Temperature and substrate conditions showed significant effects (*p* < 0.001) on archaeal communities (using ANOSIM and PERMANOVA analyses) and can explain 44% and 35% of the archaeal community variance respectively (Table S1).

The reactor operational parameters and feeding substrate characteristics (independent of temperature) were used to conduct Canonical Correspondence Analysis (CCA) to explain the variation in communities (Supplementary Fig. S1). It showed that temperature, influent substrate concentration (total COD, soluble COD, pCOD/tCOD), organic loading rate, and hydrolysis rate constant (*K*_H_) had significant correlations (*p* < 0.05, Supplementary Table S2) with bacterial community variation but not *D*_rate_ (relative difference between *K*_H_ and total SMA). The hydrolysis potential of the substrate (*K*_H_) is determined by both the substrate type and reactor temperature, showing significant correlation with the bacterial community variance (*p* < 0.05, Supplementary Table S2). The *D*_rate_ showed a significant association with archaeal community variance (*p* < 0.05, Supplementary Table S2), indicating that archaeal community may be affected more by the unbalanced substrate hydrolysis potential and methanogenic potential.

The alpha diversity indexes (Shannon diversity, Number of genera and Evenness) showed general decreasing trends with deceasing temperature (*p* < 0.05, Supplementary Table S3). Different substrate conditions also showed significant differences in all alpha diversity indexes (p < 0.05, Supplementary Table S3). Thermophilic co-digestion showed the lowest Shannon diversity and Evenness in both bacterial communities (1.42 ± 0.35, Fig. 1C) and archaeal communities (0.46 ± 0.06, Fig. 1D), and the lowest number of archaeal genera (3.25 ± 0.50). Less diverse community predominated the thermophilic co-digestion reactor with lower alpha-diversity indexes, indicating stronger selective effect and microbial competition.

Under the mesophilic conditions, the bacterial community of the low-solid-substrate reactor showed the highest Shannon diversity (3.98 ± 0.38) and number of genera (257 ± 37) (Fig. 1C). The mesophilic regular-substrate reactor showed the highest evenness (0.80 ± 0.04). For the archaeal community, the mesophilic low-solid-substrate reactor showed the highest Shannon diversity, number of genera and evenness (1.85 ± 0.33, 14 ± 1, 0.69 ± 0.12, respectively) (Fig. 1D). The differences within the mesophilic condition indicate that other factors besides temperature acted as driving forces for community diversity. The ambient reactor showed similar alpha-diversity indexes with the mesophilic low-solid-substrate reactors except for a lower number of archaeal genera (10 ± 2).

**Fig. 2:**
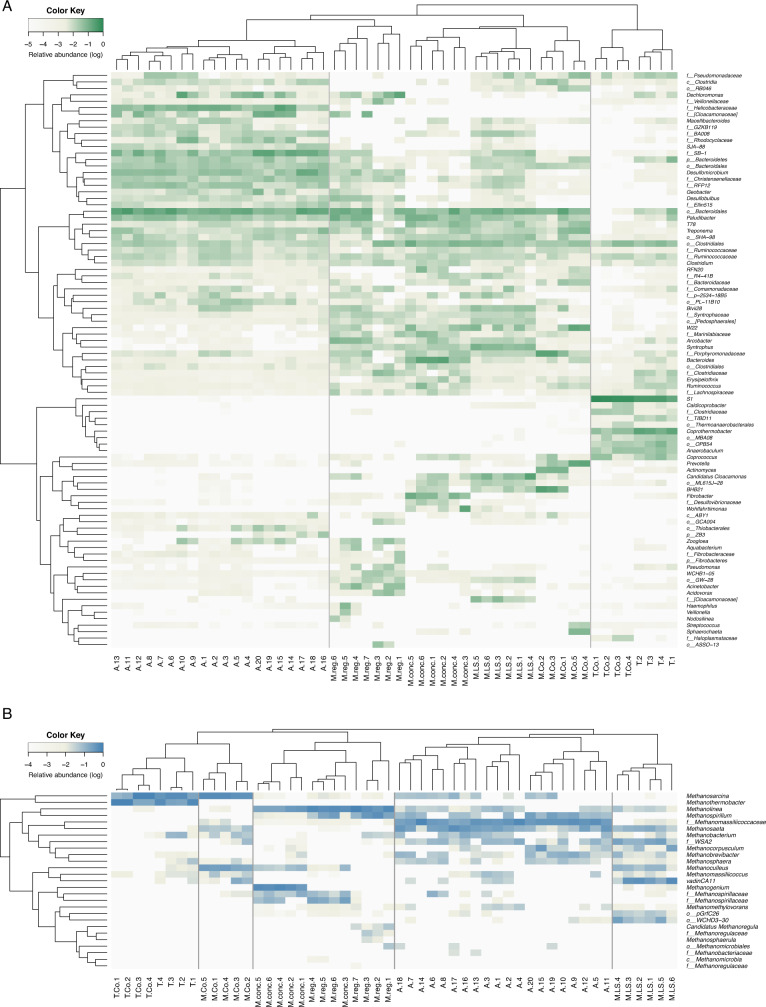
Microbial community composition. **A** Assemblage of the top 10 bacterial genera from each sample. **B** All archaeal genera. Reactor abbreviation A: Ambient; M.LS: Mesophilic Low-solid-substrate; M.reg: Mesophilic regular-substrate; M.conc: Mesophilic concentrated-substrate; M.Co: Mesophilic Co-digestion; T: Thermophilic; T.Co: Thermophilic Co-digestion. Number in sample name indicates replicates from the same group.

The top 10 most abundant bacterial genera from each sample were compared, as shown in Fig. 2A. A clear clustering pattern associated with temperature was observed at the genus level and also at the phylum level (Supplementary Fig. S2). At ambient temperature, the predominant bacteria included unidentified genera from the order *Bacteroidales* (5–36%), the family SB-1 (phylum ﻿*Bacteroidetes*) (1–25%), the family *Helicobacteraceae* (phylum ﻿*Proteobacteria*) (1–27%), the family *Christensenellaceae* (phylum ﻿*Firmicutes*) (1–4%), and *Desulfomicrobium* (phylum ﻿*Proteobacteria*) (1–17%). These bacteria were observed in the mesophilic reactors at lower relative abundances, while they disappeared or had very low abundances in the thermophilic reactors. At thermophilic conditions, the genus S1 (phylum ﻿*Thermotogae*) showed significant abundances (20–80%) in both blackwater and co-digestion reactors.

The archaeal communities formed five clusters (Fig. 2B). The thermophilic reactors and mesophilic co-digestion reactors were clustered in one group, sharing the high-abundance archaeal genus *Methanosarcina* (10–62%). The second most abundant genus differed, *Methanothermobacter* in the thermophilic reactors (19–89%), and *Methanoculleus* in the mesophilic co-digestion reactor (17–30%). The mesophilic concentrated-substrate and regular-substrate reactors shared common predominant genera, *Methanospirillum* and an unclassified genus in the order *Methanospirillaceae*. Even though all at mesophilic temperature, the low-solid-substrate reactors differed from concentrated-substrate and regular-substrate reactors. The most abundant genus was vadinCA11 (family ﻿*Methanomassiliicoccaceae*, 14–62%) for mesophilic low-solid-substrate reactor. The obligate acetoclastic methanogen *Methanosaeta* was also enriched (3–18%), but not in the other mesophilic reactors. At the ambient temperature, an unclassified genus in the family *Methanomassiliicoccaceae* (18–73%) was predominant, followed by *Methanosaeta* (1–50%), *Methanospirillum* (0–26%), *Methanobacterium* (0–17%), and an unclassified genus in the family WSA2 (0–16%). Compared to the thermophilic and mesophilic conditions, the ambient temperature reactors had higher microbial evenness (Fig. 1D), dominated by multiple genera.

Comparing the clustering patterns of bacterial and archaeal communities by PCoA (Fig. 1A, B) and by the dominant genera (Fig. 2), it is suggested that five community groups can be classified: ambient, mesophilic low-solid-substrate, mesophilic (concentrated-substrate and regular-substrate), mesophilic co-digestion, and thermophilic. These five groups were used for co-occurrence network analysis in the following sections.

### Co-occurrence network analysis

**Fig. 3:**
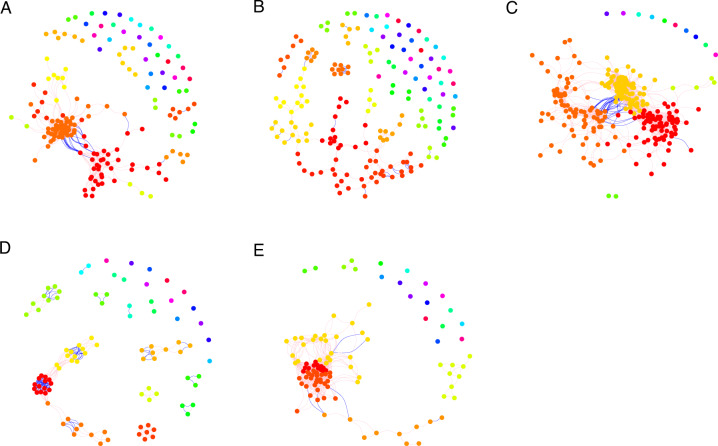
The co-occurrence network of five groups. (**A**) Ambient. (**B**) Mesophilic Low-solid-substrate. (**C**) Mesophilic (regular-substrate and concentrated-substrate). (**D**) Mesophilic Co-digestion. (**E**) Thermophilic (sole-blackwater and co-digestion). Color of nodes indicates OTUs from the same module in each network. Line color indicates positive (pink) and negative (blue) correlation coefficient. Spearman’s correlation coefficient *r* > 0.6 and *p* < 0.01 were used for network construction.

The clustering pattern of the five groups led to construction of five co-occurrence networks using the significant correlations among genera (Spearman’s correlation coefficient *r* > 0.6, *p* < 0.01). As shown in Fig. 3 and Table 2, the network characteristics varied for each group. The clustering coefficient represents the complexity of the network and strong interactions among microorganisms. The mesophilic co-digestion network showed the highest clustering coefficient (0.85), followed by the thermophilic network (0.70), indicating that microbial interactions are strongest in these two groups. A previous study showed that higher clustering coefficient corresponds to more dynamic and active community^21^. The average path length values were the lowest in these two groups (3.04 and 2.63 for mesophilic co-digestion and thermophilic condition, respectively), indicating compact network property and strong microbial interactions. These network properties can be associated to the high-hydrolysis efficiency (51.8–80.5%) and methanogenesis efficiency (51.6–77.1%) (Table 1), which were remarkably higher than at other conditions.

**Table 2:** Key characteristics of co-occurrence networks of five groups.

Group	Clustering coefficient	Random Network clustering coefficient	Modularity	Positive ratio	Average path length	Average degree	Average normalized degree	Average normalized betweenness
Ambient	0.69	0.06	0.4	0.97	3.7	10.0	0.06	0.007
Mesophilic Low-solid-substrate	0.56	0.07	0.81	0.88	7.5	2.9	0.02	0.011
Mesophilic	0.59	0.04	0.49	0.98	3.3	17.6	0.07	0.008
Mesophilic Co-digestion	0.85	0.06	0.7	0.64	3	5.6	0.06	0.004
Thermophilic	0.7	0.13	0.21	0.99	2.6	15.3	0.14	0.009

The mesophilic low-solid-substrate network presented the lowest clustering coefficient (0.56) and highest average path length (7.52). Meanwhile, it showed the highest modularity (0.81) and lowest average normalized degree (0.02), indicating less inter-dependence among the microorganisms and more divergent functional groups. This observation correlates to the substrate property with lower solids content and reduced requirement for hydrolysis (16%), leading to weaker dependence on hydrolytic bacteria by the acidogens, acetogens and methanogens compared to the other networks.

Three of the co-occurrence networks showed high ratios of positive correlations, the ambient (97%), mesophilic (98%) and thermophilic (99%) networks. Whereas the mesophilic low-solid-substrate network and the mesophilic co-digestion network showed fewer positive correlations (88% and 64%, respectively).

### Linking network characteristics and reactor parameters

**Fig. 4:**
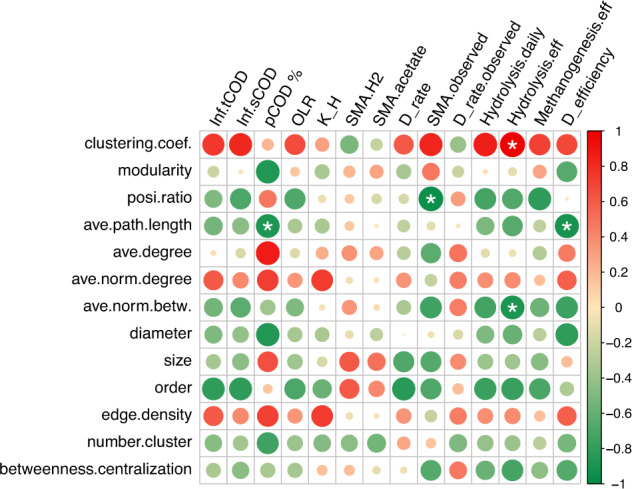
Correlation matrix between co-occurrence network characteristics and operational parameters. Color bar indicates Pearson correlation coefficient. Significance labeled (*) for *p* < 0.05.

Comparison of the co-occurrence network characteristics inferred relationships with reactor hydrolysis and methanogenesis performances and activities. Therefore, the variables related to hydrolysis and methanogenesis were tested for correlation with network characteristics among all variables (Fig. 4).

The clustering coefficient was significantly (*p* < 0.05) positively correlated with hydrolysis efficiency, while the average normalized betweenness was significantly (*p* < 0.05) negatively correlated with hydrolysis efficiency, indicating that higher hydrolysis may depend on stronger microbial interactions. The average path length was significantly (*p* < 0.05) negatively correlated with two variables, the pCOD percentage, and the *D*_efficiency_. When the pCOD percentage is high, it is required for higher hydrolytic activity and fast consumption of the hydrolyzed products, which may lead to the short network path length. The relative differential efficiency between hydrolysis and methanogenesis (*D*_efficiency_) may indicate the dependence on transportation or syntrophic production and consumption of intermediates between different functional microorganisms. Higher *D*_efficiency_ resulted in more complex network (higher clustering coefficient) and shorter average path length. Modularity showed similar patterns with average path length, but not significant for any correlation. The network positive correlation ratio showed significantly (*p* < 0.05) negative correlation with the observed SMA, a feature of the in-situ activity in the reactors.

### Keystone OTUs

**Fig. 5:**
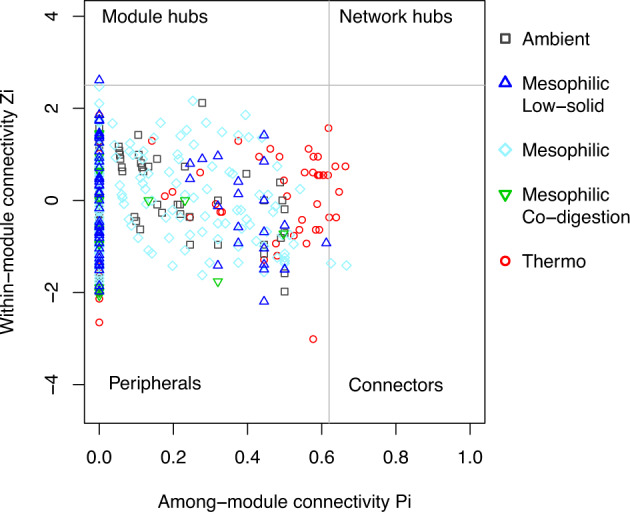
Within-module and among-module connectivities. Z_i_-P_i_ plot of the individual genera from five groups: Ambient, Mesophilic Low-solid-substrate, Mesophilic (regular-substrate and concentrated-substrate), Mesophilic Co-digestion, Thermophilic (sole-blackwater and co-digestion).

Besides the general co-occurrence network characteristics, the topological features of the individual OTUs are informative to indicate their ecological roles. As shown in Fig. 5, OTUs were classified into four groups based on the within-module connectivity (Z_i_) and among-module connectivity (P_i_)^22^. The peripherals (low Z_i_, low P_i_) are OTUs that have a few links to other OTUs within their modules, which included the majority of OTUs in all groups. The module hubs (high Z_i_, low P_i_) present OTUs with high number of links in their own modules. Only the mesophilic low-solid-substrate network had one module hub, *Roseburia* (order *Clostridiales*).

The connectors (low Z_i_, high P_i_) describe OTUs with links to several modules, inferring important roles for inter-module communication. The mesophilic and thermophilic networks showed a few connectors. In the mesophilic network, *Streptococcus* (order *Lactobacillales*) and an unclassified genus in the family *Methanospirillaceae* were classified as connectors. In the thermophilic network, five genera were classified as connectors, *Ruminofilibacter* (order *Bacteroidales*), *Paludibacter* (order *Bacteroidales*), *Fibrobacter* (order *Fibrobacterales*), *Lachnospira* (order *Clostridiales*), and an unclassified genus in the family *Anaerobaculaceae* (order *Synergistales*).

**Fig. 6:**
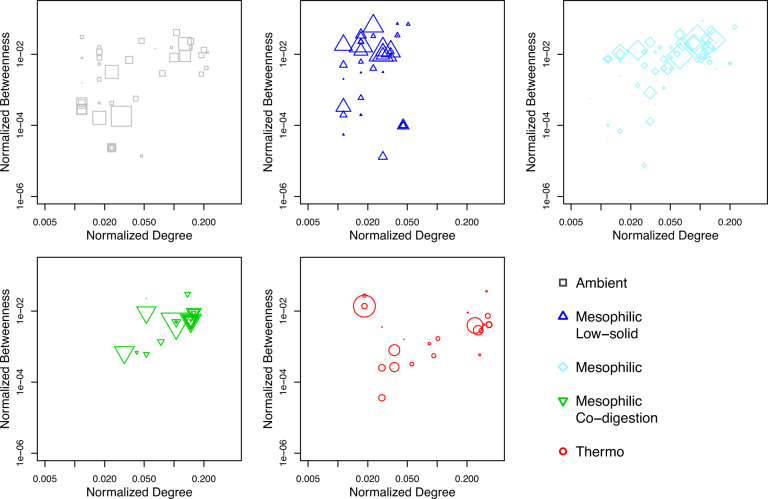
Normalized betweenness and normalized degree of individual genera. The size of symbols represents the relative abundances of genera. Five groups: Ambient, Mesophilic Low-solid-substrate, Mesophilic (regular-substrate and concentrated-substrate), Mesophilic Co-digestion, Thermophilic (sole-blackwater and co-digestion).

The normalized betweenness and degree for individual OTUs are shown in Fig. 6. The degree of an individual OTU indicates its level of interaction with other OTUs. High degree suggests a central hub role in the network. The high betweenness reveals the role for connecting other OTUs, referred as gatekeepers, suggesting the importance of maintaining communication, integrity and function of microbial communities^23^. Generally, the distribution of normalized betweenness and normalized degree showed positive relationships in all networks^21^. The mesophilic low-solid-substrate network showed lower normalized degree distribution than other networks, which is also reflected in the lowest average normalized degree (Fig. 3). The thermophilic network showed a trend of high normalized degree distribution but lower normalized betweenness than other networks, suggesting high interaction levels but lower dependence on connector OTUs, which can be reflected by the shortest average path length (Fig. 3). The relative abundances of OTUs are represented by the symbol size and showed no correlation with the distribution of normalized betweenness or degree.

The 10 highest normalized betweenness and normalized degree OTUs in each network are shown in Table 3. It can be seen that among different networks, the OTUs functioning as central hubs (highest normalized degree) were different, so were the connecting OTUs (highest normalized betweenness). Only a few OTUs appeared concurrently in different networks. The genus T78 (phylum *Chloroflexi*) had high normalized degree in both ambient and mesophilic co-digestion networks. An unidentified genus in the order GCA004 (phylum *Chloroflexi*) was shown in both mesophilic and mesophilic co-digestion networks. *Fibrobacter* (phylum *Fibrobacteres*) was in mesophilic, mesophilic co-digestion and thermophilic networks as high normalized betweenness genus. *Desulfobulbus* (phylum *Proteobacteria*) was common in mesophilic low-solid-substrate and mesophilic networks to have high normalized betweenness.

**Table 3:** The 10 highest normalized betweenness and normalized degree OTUs in each network.

	Highest Normalized Degree		Highest Normalized Betweenness	
	Phylum	Identified level	Phylum	Identified level
**Ambient**	Bacteroidetes	f__Cytophagaceae	OP3	c__koll11
	Bacteroidetes	f__Chitinophagaceae	OD1	c__ZB2
	Bacteroidetes	Niabella	TM6	c__SJA-4
	Chloroflexi	SHD-231	Firmicutes	**Fusibacter**
	Chloroflexi	T78	Spirochaetes	SJA-88
	Proteobacteria	f__Comamonadaceae	Proteobacteria	o__Burkholderiales
	Bacteroidetes	f__Saprospiraceae	Firmicutes	f__Clostridiaceae
	Proteobacteria	c__Betaproteobacteria	Bacteroidetes	f__Bacteroidaceae
	Firmicutes	**Fusibacter**	Crenarchaeota	o__pGrfC26
	Proteobacteria	Simplicispira	FCPU426	FCPU426
**Meso Low solids**	Bacteroidetes	**Macellibacteroides**	Proteobacteria	f__Desulfobacteraceae
	Bacteroidetes	Paludibacter	Firmicutes	Faecalibacterium
	Bacteroidetes	Parabacteroides	Spirochaetes	f__Spirochaetaceae
	Lentisphaerae	Victivallis	Bacteroidetes	f__Cryomorphaceae
	Lentisphaerae	f__R4-45B	Planctomycetes	c__Phycisphaerae
	Verrucomicrobia	f__R4-41B	Firmicutes	Roseburia
	Bacteroidetes	f__Rikenellaceae	Proteobacteria	Desulfobulbus
	Bacteroidetes	f__p-2534-18B5	Proteobacteria	Desulfomicrobium
	Chloroflexi	o__GCA004	Bacteroidetes	**Macellibacteroides**
	Firmicutes	RFN20	Euryarchaeota	Methanosaeta
**Meso**	Proteobacteria	**Acidovorax**	Tenericutes	o__ML615J-28
	Synergistetes	PD-UASB-13	Proteobacteria	**Acidovorax**
	Euryarchaeota	f__Methanoregulaceae	Firmicutes	**Anaerovorax**
	Chloroflexi	o__GCA004	Proteobacteria	Desulfobulbus
	Firmicutes	Carnobacterium	Firmicutes	f__Tissierellaceae
	Firmicutes	f__Peptostreptococcaceae	Proteobacteria	Wohlfahrtiimonas
	Chlorobi	c__SJA-28	Proteobacteria	Sulfuricurvum
	Proteobacteria	Shinella	Proteobacteria	o__Myxococcales
	Proteobacteria	f__Sphingomonadaceae	Fibrobacteres	Fibrobacter
	Firmicutes	**Anaerovorax**	Cyanobacteria	o__YS2
**Meso Co-digestion**	Bacteroidetes	**Petrimonas**	Fibrobacteres	Fibrobacter
	Chloroflexi	T78	Euryarchaeota	f__Methanomassiliicoccaceae
	Proteobacteria	Geobacter	Firmicutes	Tissierella Soehngenia
	Proteobacteria	Aeromonas	Bacteroidetes	Proteiniphilum
	Proteobacteria	f__Pseudomonadaceae	Firmicutes	Pseudobutyrivibrio
	Spirochaetes	Treponema	Firmicutes	vadinHB04
	Euryarchaeota	Methanobacterium	Synergistetes	vadinCA02
	Euryarchaeota	vadinCA11	WWE1	W22
	Bacteroidetes	f__Marinilabiaceae	WWE1	W5
	Bacteroidetes	f__Porphyromonadaceae	Bacteroidetes	**Petrimonas**
**Thermo**	Firmicutes	Proteiniclasticum	Firmicutes	f__Planococcaceae
	Planctomycetes	o__MSBL9	Firmicutes	Defluviitalea
	Proteobacteria	o__Campylobacterales	Firmicutes	Symbiobacterium
	Firmicutes	Butyrivibrio	Tenericutes	o__ML615J-28
	Firmicutes	**Sedimentibacter**	Firmicutes	c__OPB54
	Firmicutes	SMB53	Bacteroidetes	Paludibacter
	Proteobacteria	Agrobacterium	Firmicutes	Caldicoprobacter
	Actinobacteria	Actinomyces	Proteobacteria	Pusillimonas
	Bacteroidetes	Bacteroidetes	Firmicutes	**Sedimentibacter**
	Bacteroidetes	o__Bacteroidales	Fibrobacteres	Fibrobacter

Some methanogens exhibited important roles in the networks (Table 3), i.e., *f_Methanoregulaceae*, and *Methanobacterium* showed high normalized degree while *Methanosaeta* and *f_Methanomassiliicoccaceae* showed high normalized betweenness. *Methanosaeta* is an obligate acetoclastic methanogen while the others are hydrogenotrophic. They are syntrophic partners of many acetogens and acidogenic (H_2_-producing) bacteria^5^, exhibiting many links in the network.

Within each network, the central hubs and the connectors only shared 1–2 same OTUs. *Fusibacter* in the ambient network, *Macellibacteroides* in the mesophilic low-solid-substrate network, *Acidovorax* and *Anaerovorax* in the mesophilic network, *Petrimonas* in the meso co-digestion network, and *Sedimentibacter* in the thermophilic network had dual network functions.

## Discussion

Operational conditions have been demonstrated to affect microbial communities in AD systems but whether the dynamics of bacterial and archaeal communities synchronize or differ is not clear. Previous full-scale AD survey studies have included multiple factors and demonstrated that substrate type (e.g., sewage sludge, manure, agricultural wastes) and temperature drove microbial community diversity, and demonstrated covariation of bacteria and archaeal communities^15,20,24^. Given the complexity of the substrate composition and large differences between substrates, pooling different types of substrates in one analysis may underestimate other factors, and conceal the variation between bacterial and archaeal responses to operational factors. Our analysis focused mainly on blackwater, while covered a wide range of factors for inter-reactor comparison. Even though it has been recognized that bacterial and archaeal communities are linked through syntrophic partnerships, we observed that their composition and diversity rely on different factors based on the Variation Partition Analysis (VPA, Supplementary Fig. S3).

Temperature is the most significant factor affecting the bacterial community based on VPA (27% of total variance, Supplementary Fig. S3), which has been reported in other studies^15,20^. Other factors such as substrate type^15,20^, organic loading rate, solids content^5,11^, and free ammonia^1,20^ can affect the bacterial community. While it may be simple and reliable to focus on one operational factor at a time, the practical application of AD often faces multiple influencing factors. For single-factor analysis, the bacterial and archaeal communities may exhibit synchronized changes along the axis of the factor, and the enriched bacteria and archaea can indicate their selection advantages and covariant dynamics. Whereas under multiple factor variations, the selection mechanism and bacteria-archaea interaction are more complex. Our study evaluated multiple factors and underlined the remarkable influence of temperature compared to all other factors. It was interesting that with the same type of substrate feed (i.e., blackwater), other factors other than temperature exhibited higher influence on archaeal communities compared to on bacterial communities as shown in Figs. 1 and 2, and VPA analysis (Supplementary Fig. S3). Influent total COD contributed to slightly higher level of variance than temperature (27.2% and 26.6% respectively in VPA). Especially, the *D*_rate_ could explain 21% of archaeal community variance and only 10% of bacterial community variance. This phenomenon has not been emphasized or investigated although some hints have been observed in a full-scale study with complex substrates^15^. Another full-scale AD study on primary and secondary sludge showed contrary conclusion^25^, where the archaeal community was more affected by temperature than the bacterial community; however, it should be noticed that the substrate feed was mainly mixed primary sludge and waste activated sludge which can vary considerably among different reactors. Our results showed that different temperatures (thermophilic and mesophilic co-digestion) may enrich the same methanogen (*Methanosarcina*), which probably relates to the easy-to-hydrolyze conditions for the substrate.

Using co-occurrence network analysis on groups divided based on the community clustering pattern, different topological characteristics can be observed which may imply links to bioreactor performances and microbial ecological features. Especially, some characteristics of the separated networks (i.e., clustering coefficient, average path length, normalized betweenness) were correlated with rector performances (Fig. 4) and have the potential to link and predict system function or efficiency. In our study, higher clustering coefficients and shorter average path lengths were features of the high-hydrolysis-rate and high-methanogenesis-rate groups, i.e., thermophilic and mesophilic co-digestion (Table 2). Although the number of separated network studies is limited, this approach is promising to show that the topological characteristics of co-occurrence networks are indicative of the ecological features of microbiomes inferred by our results and other microbiome network studies. In comparison to soil microbiome networks, the clustering coefficient and connectedness showed increases in early recovery and decreases in late recovery after disturbance^21^, indicating that weaker interaction (smaller clustering coefficient) predicts higher community stability^26^. In a co-occurrence network study of steady-state activated sludge^17^, small clustering coefficient and short average geodesic distance (average path length) were linked with the optimal substrate biodegradability condition and the BOD removal load, indicating generally higher microbial activity. It should be noted that AD systems contain more microbial interactions for cooperation and syntrophic partnership compared with aerobic systems; and the average clustering coefficient (0.59–0.85) in our study was much higher than the activated sludge networks (0.059–0.402)^17^ and soil bacterial networks (0.08–0.24)^21^. Comparison of the AD networks in our study demonstrated that stronger interaction (higher clustering coefficient and shorter average path) is linked to higher rates and activities of microbial communities under steady-state conditions. It should be noted that the implication to stability is not included in the present study, which can be tested using non-steady-state bioreactors in future studies.

The high modularity indicates the formation of sub-communities, representing high niche differentiation in other ecosystems^17^. In activated sludge networks, the modularity was indicated to reflect parallel functional groups formed under desirable (non-stressful, less limitation) and less-disturbance conditions, while interaction and dependency between microbes are less required compared to high-disturbance conditions^17^. Cooperative niches are essential for AD system performance rather than parallel niches. In reported AD systems, the modularity was positively correlated with methane production^12^, while the clustering coefficient was not reported, thus not able for comparison with our study. Another AD study demonstrated multiple networks for sewage sludge and food waste co-digestion without comparing the network topological characteristics^16^. Nevertheless, it showed network differences from non-stable to stable status, inferring that the network characteristics may indicate system stability.

Even though the clustering coefficient and the modularity showed different meanings for different types of microbiomes (soil, activated sludge and AD), the average path length seems to have a global meaning. Shorter path length is correlated with higher efficiencies of BOD and nutrient removal in activated sludge^17^, and negatively with particulate COD percentage and relative differential efficiency between hydrolysis and methanogenesis (*D*_efficiency_) in AD in our study. The ecological implication of shorter path length can link to better microbial cooperation, more communication and intermediate transport, which are crucial for AD microbiome.

The topological characteristics of individual nodes (OTUs) are indicative of the ecological roles of the OTUs. For example, *Roseburia*, the module hub in mesophilic low-solid-substrate network, is a butyrate-producing anaerobic bacterial genus^27^ and may perform acidogenesis that connects hydrolysis and downstream acetogenesis and methanogenesis. The approach of identifying keystone OTUs from network analysis is therefore useful to bring insights to understand the biochemical functions and microbial interactions. Identifying keystone OTUs using Z_i_-P_i_ (within-module connectivity and among-module connectivity) plot has been used in many studies^17,22,28^, although it is also recognized that the majority of OTUs were peripherals^17^ and lacked ecological implication. The betweenness and degree of nodes are informative features to indicate OTUs’ ecological roles as communication connectors and central hubs^21^. Noticeably, these important roles are not correlated with the relative abundance of the OTUs (Fig. 6). The high-abundance OTUs are functionally important for reactors’ performances but not always perform central roles in the network^17,22,28^.

Lower-abundance OTUs (as low as 0.1%) can be found to have high normalized betweenness and degree, indicating their important roles in maintain the system’s ecological stability and functionality. These OTUs differed in the five AD networks (Table 3), underlining the ecological importance of OTUs with low abundance and high betweenness and degree in different systems and potential to use them as ecological indicators. T78 (ambient and mesophilic co-digestion networks) was reported in anaerobic digesters and suggested to produce acetic acid (acetogenesis) from long-chain fatty acids^29^, which explains its central hub role linking with many bacteria and archaea since acetic acid can be utilized widely. *Fibrobacter* (mesophilic, mesophilic co-digestion and thermophilic networks) is a common cellulose-degrading genus in AD^30^, which is likely to perform cellulose hydrolyzation from the feedstock and supply metabolites to acidogenetic and acetogenetic bacteria with high efficiency. *Desulfobulbus* (mesophilic low-solid-substrate and mesophilic networks) can oxidize propionate with sulfate to produce acetate^31^, linking the propionate-producing bacteria and acetate-consuming bacteria in mild conditions.

Among the operational factors, influent substrate characteristics influenced archaeal communities in addition to temperature which affected both bacterial and archaeal communities. Moreover, the network topological properties were correlated with influent substrate characteristics. Higher hydrolysis efficiency of the substrate associated with higher clustering coefficient and lower normalized betweenness of the network, indicating higher complexity and microbial interactions. One of the network’s topological characteristics, average path length, can be an indicator of system performance, linking the network model to engineering operations. Individual OTUs’ topological characteristics showed that (i) high-rate thermophilic reactor group had more connector OTUs (e.g., *Ruminofilibacter, Paludibacter*), inferring stronger inter-module communication, and (ii) many lower-abundance OTUs (as low as 0.1%) may be important members to maintain the stability and functionality of the microbial community. This study differentiates bacterial and archaeal communities’ driving factors, reveals the links between system performance and network features, and raises our attention on ecological importance of lower-abundance OTUs (as low as 0.1%) besides the top ranked OTUs. Future work is required to expand the sampling size of full-scale systems if to apply this approach to engineering systems, e.g., real-time data acquisition and analysis could be implemented to predict, monitor and troubleshoot the system performance. Low-abundance taxa’s ecological roles should be investigated using integrated approaches on top of network topology.

## Methods

### Reactors

Anaerobic digestion of blackwater was conducted under temperature ranging from 22 to 55 °C, including ambient (22 °C, 5 reactors), mesophilic (35 °C, 5 reactors) and thermophilic (52–55 °C, 2 reactors) conditions. Details of the feedstock preparation are provided in Supplementary information. Table 4 shows the reactor processes and operational parameters of 12 reactors all fed with blackwater at different concentrations (influent total chemical oxygen demand [COD]). The ambient reactors (A) used regular-substrate condition. The mesophilic reactors include different substrate conditions (low-solid-substrate [M.LS], regular-substrate [M.reg], concentrated-substrate [M.conc], and co-digestions with food waste [M.Co]). The thermophilic reactors include blackwater only [T] and co-digestions with food waste [T.Co]. Reactors were operated with increasing organic loading rate (OLR) from the initial stage to the final stage. At the end of each stage, sludge sample was completely mixed to ensure homogeneity and then collected for DNA analysis, resulting in a collection of 52 samples.

**Table 4:** Reactor processes and operational parameters.

Reactor description	Type of reactor^a^	Sample number	Volume (L)	Temperature (^o^C)	OLR^b^ (g/L/d)	Inf. tCOD (g/L)	Inf. sCOD (g/L)	SRT^c^ (d)
Ambient (A)	5 SBRs	20	1	22	0.20–0.50	1.0 (0.0)	0.3 (0.0)	NA
Mesophilic Low-solid-substrate (M.LS)	2 UASBs	6	5	35	0.35–2.25	3.7 (0.7)	2.3 (0.2)	179 (99)
Mesophilic regular-substrate (M.reg)	1 UASB	7	5	35	0.18–1.06	1.0 (0.0)	0.3 (0.0)	384 (237)
Mesophilic concentrated-substrate (M.conc)	1 UASB	6	5	35	0.28–4.07	9.9 (0.4)	3.2 (0.3)	245 (16)
Mesophilic Co-digestion^d^ (M.Co)	1 SBR	5	2	35	1.70–4.80	32.5 (1.0)	14.7 (1.0)	34 (9)
Thermophilic (T)	1 UASB	4	1	52	0.90–3.40	29.8 (7.3)	4.7 (0.9)	15 (0)
Thermophilic Co-digestion^d^ (T.Co)	1 SBR	4	2	55	1.70–3.10	32.9 (0.9)	15.3 (2.6)	30 (10)

^a^SBR, sequencing batch reactor; UASB, up-flow anaerobic sludge bed reactor.

^b^OLR, organic loading rate.

^c^SRT, solids retention time.

^d^Co-digestion is the blackwater and food waste at a volatile solid mixing ratio of 1:2.

### Chemical analysis

The COD, total solids, and volatile suspended solid (VSS) were measured using the Standard Methods 5220C, 2540B, 2540E (APHA/AWWA/WEF, 2012). pH was measured using B40PCID pH meter (VWR, SympHony). Samples were filtered through 0.45 um membrane filters (Fisher Scientific, CA) to test soluble COD, volatile fatty acids (VFAs), and total ammonia nitrogen (TAN). The VFA acetate, propionate and butyrate were measured using ionic chromatography (DIONEX ICS-2100, ThermoFisher, USA). TAN was measured using the Nessler method (HACH, Loveland, CO). Biogas samples were analyzed using 7890B gas chromatograph (Agilent Technologies, Santa Clara, USA) equipped with a thermal conductivity detector (TCD).

The specific methanogenic activity (SMA) of the reactor sludge at the end of each operation stage was measured in batch bottles fed with H_2_&CO_2_ (80%/20%) or sodium acetate, to evaluate the maximum activity according to the methods described previously^2,4^. The observed specific methanogenic activity was calculated based on the real CH_4_ production in the reactor.

### Derived parameter calculations

Free ammonia (FA) was calculated using TAN, pH and temperature (unit K).

1 $${\rm{NH}}_3(FA) = 1.214 \times {\rm{TAN}} \times \left( {1 + \frac{{10^{ - pH}}}{{10^{ - (0.09018 + \frac{{2729.92}}{T})}}}} \right)^{ - 1}$$

Hydrolysis efficiency, methanogenesis efficiency and hydrolysis rate constant (*K*_H_) were calculated using the following equations:

2 $${\rm{Hydrolysis}}\;{\rm{efficiency}}\;\left( {{{\mathrm{\% }}}} \right) = \frac{{{\rm{sCOD}}_{{\rm{eff}}} - {\rm{sCOD}}_{{\rm{inf}}} + {\rm{COD}}_{{\rm{CH4}}}}}{{{\rm{tCOD}}_{{\rm{inf}}} - {\rm{tCOD}}_{{\rm{eff}}}}}$$

3 $${\rm{Methanogenesis}}\;{\rm{efficiency}}\;\left( {{{\mathrm{\% }}}} \right) = \frac{{{\rm{COD}}_{{\rm{CH4}}}}}{{{\rm{tCOD}}_{{\rm{inf}}} - {\rm{tCOD}}_{{\rm{eff}}}}}$$

4 $$K_{\rm{H}} = \frac{1}{{{{{\mathrm{{\Delta}}}t}}}}\ln (\frac{{{\rm{COD}}_{{\rm{CH4}}\_{\rm{end}}} + {\rm{sCOD}}_{{\rm{end}}} - {\rm{sCOD}}_0}}{{({\rm{COD}}_{{\rm{CH4}}\_{\rm{end}}} + {\rm{sCOD}}_{{\rm{end}}}) - ({\rm{COD}}_{{\rm{CH4}}_t} + {\rm{sCOD}}_t)}})$$

To describe the difference between hydrolysis and methanogenesis, two variables were derived. The relative differential efficiency (*D*_efficiency_) calculates the relative difference between hydrolysis and methanogenesis efficiency. The relative differential rate (*D*_rate_) calculates the relative difference between hydrolysis rate constant (which reflects the easiness of the substrate to be hydrolyzed) and total SMA.

5 $$D_{{\rm{efficiency}}} = \frac{{{\rm{Hydrolysis}}\;{\rm{efficiency}}\;\left( {{{\mathrm{\% }}}} \right) - {\rm{Methnogenesis}}\;{\rm{efficiency}}\;({{{\mathrm{\% }}}})}}{{{\rm{Hydrolysis}}\;{\rm{efficiency}}\;\left( {{{\mathrm{\% }}}} \right)}}$$

6 $$D_{{\rm{rate}}} = \frac{{K_{\rm{H}} - {\rm{SMA}}}}{{K_{\rm{H}}}}$$

### DNA extraction and 16S rRNA gene sequencing

Genomic DNA was extracted from each sludge sample using DNeasy PowerSoil Kit (QIAGEN, Canada) following the manufacturer’s protocol. The quality and concentration of the extracted DNA were examined using gel electrophoresis and NanoDrop One (ThermoFisher, USA). PCR was performed to amplify the 16S rRNA genes using the universal primer-pair 515 F(5′-GTG CCA GCM GCC GCG GTA A-3′) and 806R(5′-GGA CTA CHV GGG TWT CTA AT-3′). The M.reg and M.conc DNA samples were amplified using bacterial primer set 357wF (5′-CCT ACG GGN GGC WGC AG-3′) and 785R (5′-GAC TAC HVG GGT ATC TAA TCC-3′) and archaeal primer set 517F (5′-GCY TAA AGS RNC CGT AGC-3′) and 909R (5′-TTT CAG YCT TGC GRC CGT AC-3′). The amplicons were sequenced on Illumina Miseq platform. The 16S rRNA amplicon sequence files are deposited in NCBI GenBank (BioProject: PRJNA730222, PRJNA701935, PRJNA639651).

### Data analysis

The demultiplexed paired raw sequences were quality-filtered and aligned using the Qiime2 pipelines^32^ “DADA2” algorithm^33^ with 99 % similarity in reference to the Greengenes (13_8). Microbial community Alpha and Beta diversities, Principal Coordinates Analysis (PCoA), Canonical Correspondence Analysis (CCA), environmental factor fitting to ordination (envfit), Variation Partition Analysis (VPA), ANOSIM and PERMANOVA were analyzed using the “vegan” package^34^ in RStudio version 3.4.1^35^. The PCoA was performed using Bray-Curtis distance. The top 10 highest relative abundance bacterial genera and all archaeal genera were plotted to heatmap using “heatmap.2” function in “gplots”.

### Co-occurrence network analysis

Co-occurrence network analysis was conducted using R “igraph”^36^ and “psych” packages^37^. Grouping of the samples into five groups resulted in different number of samples (Table S5) and the highest sample number was 20 in the Ambient group which was reduced to 8 selecting those at AD steady state at the Ambient condition for network analysis to reduce bias of large sample number. Only OTUs with relative abundance higher than 0.1% and occurred in more than two samples were included for analysis. The Spearman’s correlations at *r* > 0.6 and *p* < 0.01 were used for network construction. Although the sample number varied, it did not show significant correlation with network properties except for the size of network (Fig. S4). The size of network was not significantly correlated with other properties of the networks or reactor parameters (Figs. 4 and S4). We used the same cut-off value of correlation coefficient for all networks (*r* > 0.6) instead of varied cut-off values^38^ to be consistent among networks and to be comparable with literature^21^. The power-law fit of degree showed significance level of *p* < 0.05 for all networks except for the thermophilic network (Supplementary Table S4). Random Networks showed much lower clustering coefficients than the constructed networks (Table 2), including the thermophilic network (0.13 vs. 0.70).

The network properties, clustering coefficient, modularity, average path length, average normalized degree, and positive ratio, were analyzed using the “igraph” package. The key characteristics are shown in Table 2 and additional information in Table S5. Although the node numbers (Order, Table S5) varied, the correlation matrix did not show any significant correlations between the network properties and the node number (Fig. S4).

The reactor variables include influent substrate characteristics (tCOD and sCOD concentrations), the ratio of particulate COD in total influent COD (pCOD %, which reflects the demand for substrate hydrolysis), the hydrolysis potential of the substrate (hydrolysis rate constant, *K*_H_), SMA, observed SMA (methanogenesis activity), hydrolysis efficiency, methanogenesis efficiency, and the derived variables relative differential efficiency (*D*_efficiency_) and relative differential rate (*D*_rate_). Two connectivity types were calculated and compared, within-module connectivity (Z_i_), and among-module connectivity (P_i_)^22^. The P_i_ and Z_i_ were calculated following the previously reported method^28^. R code is available at https://github.com/bing-g/AD-BW-network.git.

### Reporting summary

Further information on research design is available in the Nature Research Reporting Summary linked to this article.

## Supplementary information

Supplemental Material

Reporting Summary

## Data Availability

The 16S rRNA amplicon sequence files are deposited in NCBI GenBank (BioProject: PRJNA730222, PRJNA701935, PRJNA639651). R code is available at https://github.com/bing-g/AD-BW-network.git.
