# Supplementary material for: Microbial co-occurrence network topological properties link with reactor parameters and reveal importance of low-abundance genera
Source: NPJ Biofilms Microbiomes. 2022 Jan 17;8:3. doi: 10.1038/s41522-021-00263-y (PMC8764041; doi:10.1038/s41522-021-00263-y)
Supplement: Supplementary file 1 — Supplemental Material [file 41522_2021_263_MOESM1_ESM.pdf]

Microbial Co-occurrence Network Topological Properties Links with Reactor Parameters and Reveals Importance of Low-abundance Genera

Bing Guo[1,2], Lei Zhang[1], Huijuan Sun[1], Mengjiao Gao[1], Najiaowa Yu[1], Qianyi Zhang[1], Anqi Mou[1] and Yang Liu[1,*]

[1] Department of Civil and Environmental Engineering, University of Alberta, Edmonton, Alberta, T6G 1H9, Canada

[2] Centre for Environmental Health and Engineering (CEHE), Department of Civil and Environmental Engineering, University of Surrey, Guildford GU2 7XH, United Kingdom

[*] Corresponding Author: Y Liu, Department of Civil and Environmental Engineering, University of Alberta, 7-263 Donadeo Innovation Centre for Engineering, Edmonton, Alberta, T6G 1H9, Canada Tel: (780) 492-5115 E-mail: yang.liu@ualberta.ca

This supplementary material contains 9 pages, 4 figures and 5 tables.

Methods

Fresh blackwater was collected from >20 persons weekly at the University of Alberta campus (Edmonton, Canada) and mixed to homogenize the feedstock. Food waste (containing vegetables, fruits, fat, oil, and grease) was collected from a food court on the same campus and grinded into small particles. The two feedstocks were stored at 4 ^{o}C before adding to reactors. For each reactor conditions, the feedstocks were diluted with tap water to reach the designed influent total COD (Table 4. Reactor processes and operational parameters) or mixed (at VS ratio of 1:2 of blackwater and food waste).

Results

Supplementary Table S1. PERMANOVA analysis of temperature and substrate effects on bacterial and archaeal communities.

		Degree of freedom	Sum of Squares	R2	F	Pr(>F)
Bacteria	Temperature	2	5.8226	0.43446	28.3708	0.001
	Substrate	4	2.9616	0.22098	7.2152	0.001
	Residual	45	4.6177	0.34456		
	Total	51	13.4018	1		
Archaea	Temperature	2	7.472	0.44022	46.438	0.001
	Substrate	4	5.8811	0.34649	18.275	0.001
	Residual	45	3.6203	0.21329		
	Total	51	16.9734	1		

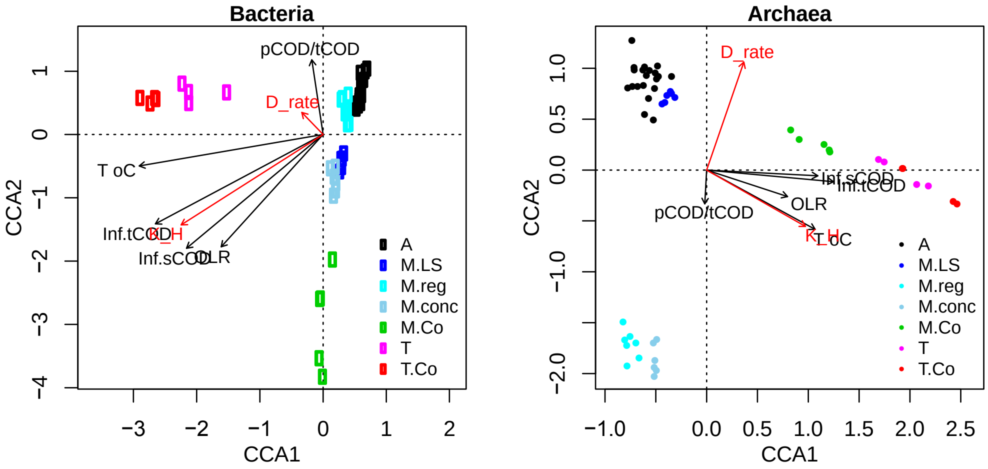

Supplementary Figure S1. Canonical Correspondence Analysis (CCA) using operational and substrate characteristics to explain the variation in communities. OLR, organic loading rate. Reactor description: Ambient (A), Mesophilic Low-solid-substrate (M.LS), Mesophilic regular-substrate (M.reg), Mesophilic concentrated-substrate (M.conc), Mesophilic Co-digestion (M.Co), Thermophilic (T), Thermophilic Co-digestion (T.Co). Same sample symbols indicate replicates from the same group.

Supplementary Table S2. Correlation between abiotic factors and CCA ordination axis.

		CCA1	CCA2	r2	Pr(>r)
Bacteria	Temperature	-0.973	-0.230	0.820	**0.001**
	pCOD/tCOD	-0.075	0.997	0.155	**0.020**
	OLR	-0.670	-0.742	0.626	**0.001**
	Inf.tCOD	-0.862	-0.507	0.915	**0.001**
	Inf.sCOD	-0.755	-0.655	0.837	**0.001**
	K_H	-0.824	-0.567	0.730	**0.001**
	D_rate	-0.677	0.736	0.022	0.596
Archaea	Temperature	0.882	-0.472	0.848	**0.001**
	pCOD/tCOD	-0.021	-1.000	0.062	0.221
	OLR	0.948	-0.319	0.398	**0.001**
	Inf.tCOD	0.993	-0.119	0.879	**0.001**
	Inf.sCOD	0.997	-0.082	0.669	**0.001**
	K_H	0.874	-0.486	0.717	**0.001**
	D_rate	0.317	0.948	0.685	**0.001**

Supplementary Table S3. ANOVA test on alpha diversity indexes with different temperature and substrate conditions.

Bacteria					
	Shannon				
	Df	SumOfSqs	mean sqs	F	Pr(>F)
Temperature	1	9.5827	9.5827	104.249	**2.70E-13**
Substrate	5	13.6177	2.7235	29.629	**3.47E-13**
Residuals	45	4.1365	0.0919		
	No.of genera				
	Df	SumOfSqs	mean sqs	F	Pr(>F)
Temperature	1	125968	125968	77.579	**2.37E-11**
Substrate	5	121709	24342	14.991	**1.17E-08**
Residuals	45	73068	1624		
	Evenness				
	Df	SumOfSqs	mean sqs	F	Pr(>F)
Temperature	1	0.16263	0.162631	86.136	**5.11E-12**
Substrate	5	0.64453	0.128906	68.273	**2.20E-16**
Residuals	45	0.08496	0.001888		

Archaea					
	Shannon				
	Df	SumOfSqs	mean sqs	F	Pr(>F)
Temperature	1	4.8984	4.8984	88.0523	**3.67E-12**
Substrate	5	2.711	0.5422	9.7466	**2.34E-06**
Residuals	45	2.5034	0.0556		
	No. of genera				
	Df	SumOfSqs	mean sqs	F	Pr(>F)
Temperature	1	64.936	64.936	23.061	**1.77E-05**
Substrate	5	259.332	51.866	18.42	**6.43E-10**
Residuals	45	126.712	2.816		
	Evenness				
	Df	SumOfSqs	mean sqs	F	Pr(>F)
Temperature	1	0.57835	0.57835	82.3591	**9.93E-12**
Substrate	5	0.21411	0.04282	6.0979	**0.0002159**
Residuals	45	0.31601	0.00702		

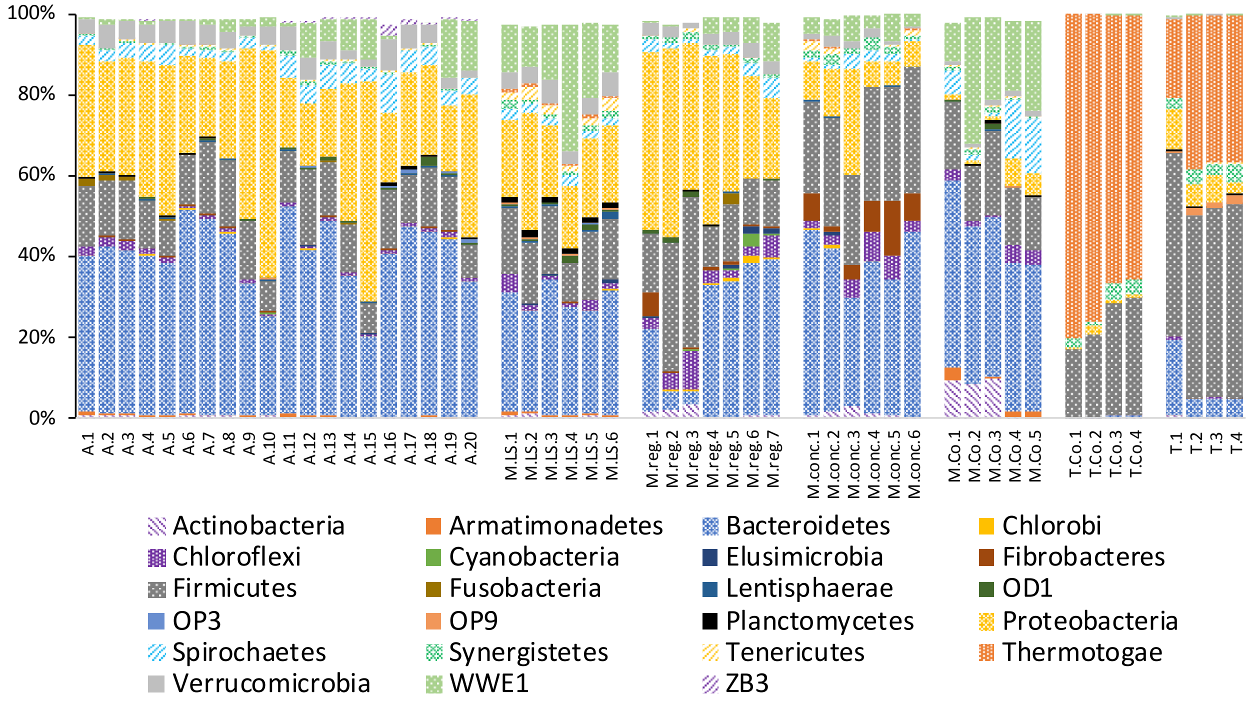

Supplementary Figure S2. Bacterial phyla with relative abundance >1% in any sample. Ambient (A), Mesophilic Low-solid-substrate (M.LS), Mesophilic regular-substrate (M.reg), Mesophilic concentrated-substrate (M.conc), Mesophilic Co-digestion (M.Co), Thermophilic (T), Thermophilic Co-digestion (T.Co). Number indicates replicates from the same group.

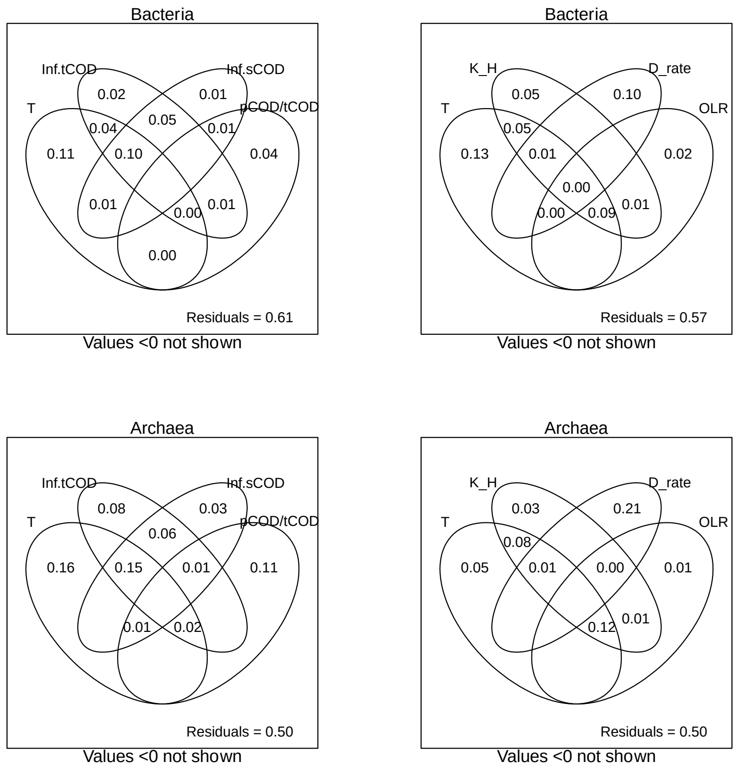

Supplementary Figure S3. Variation partition analysis (VPA) of bacterial and archaeal communities with reactor variables.

Supplementary Table S4. Power-law fit of degree results of the five networks.

	Ambient	Mesophilic Low-solid-substrate	Mesophilic	Mesophilic Co-digestion	Thermophilic
Continuous	TRUE	TRUE	TRUE	TRUE	TRUE
alpha	1.54	2.54	2.43	2.50	10.70
xmin	0.006	0.011	0.047	0.042	0.315
logLik	239	361	258	105	47
KS.stat	0.182	0.248	0.150	0.219	0.176
KS.p	1.54E-04	2.18E-06	0.002	0.009	0.536

Table S5. Additional summary of the network properties.

Group	Diameter	Size	Order	Edge density	No. of cluster	Betweenness centralization
Ambient	13	851	170	0.059	37	0.078
Mesophilic Low-solid-substrate	21	251	176	0.016	48	0.113
Mesophilic	9	2252	256	0.069	12	0.079
Mesophilic Co-digestion	10	268	95	0.060	24	0.052
Thermophilic	10	825	108	0.143	18	0.101

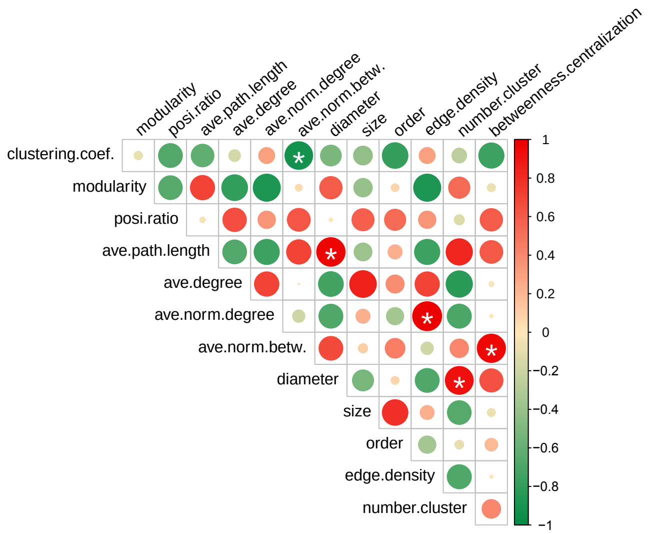

Supplementary Figure S4. Correlation matrix between the network characteristics. Color bar indicates Pearson correlation coefficient. Significance labelled (*) for p <0.05.
